# Elevated expressions of survivin and VEGF protein are strong independent predictors of survival in advanced nasopharyngeal carcinoma

**DOI:** 10.1186/1479-5876-6-1

**Published:** 2008-01-03

**Authors:** Yu-Hong Li, Chun-Fang Hu, Qiong Shao, Ma-Yan Huang, Jing-Hui Hou, Dan Xie, Yi-Xin Zeng, Jian-Yong Shao

**Affiliations:** 1 State Key Laboratory of Oncology in Southern China, Guangzhou 510060, PR China; 2 Department of Medical Oncology, Sun Yat-Sen University Cancer Center, Guangzhou 510060, PR China; 3 Department of Pathology, Sun Yat-Sen University Cancer Center, Guangzhou 510060, PR China; 4 Department of Experiment Research, Sun Yat-Sen University Cancer Center, Guangzhou 510060, PR China

## Abstract

**Background:**

Nasopharyngeal carcinoma (NPC) is one of the most common malignancies in southern China. The China 1992 TNM staging system has been widely used for prognosis prediction of NPC patients in China. Although NPC patients can be classified according to their clinical stage in this system, their prognosis may vary significantly.

**Method:**

280 cases of NPC with clinical follow-up data were collected and expressions of survivin and VEGF in tumor tissues were investigated by immunohistochemistry (IHC). Apoptosis index (AI) in 100 cases of NPC was detected by the TUNEL method.

**Results:**

Expression of survivin and VEGF were significantly associated with TNM stage, T-stage and metastasis of NPC. The patients with survivin and VEGF over-expression presented lower 5-year survival rate, as compared to those of low-expression (42.32% vs. 70.54%, 40.1% vs. 67.8%, respectively, *P *< 0.05), especially in advanced stage patients (36.51% vs. 73.41%, 35.03% vs. 65.22%, respectively, *P *< 0.05). The 5-year survival rate in NPC patients with survivin and VEGF dual over-expression was significantly lower than that of patients with dual low-expression (18.22% vs. 73.54%, respectively; *P *= 0.0003). Multivariate analysis indicated that both survivin and VEGF over-expression in NPC tumor tissues were strong independent factors of poor prognosis in NPC patients. The mean AI in the 39 survivin low-expression cases was 144.7 ± 39.9, which was significantly higher than that in 61 survivin over-expression cases (111.6 ± 39.8) (T test, P < 0.05).

**Conclusion:**

Survivin and VEGF over-expression are independent prognostic factors for the patients with NPC. These results also suggest that tumor survivin and VEGF expressions are valuable prognostic markers for prognosis prediction in NPC patients.

## Introduction

Inhibition of apoptosis may be involved in the pathogenesis of cancer by prolonging cell life and facilitating retention of deleterious mutations. Several inhibitors of apoptosis related to the baculovirus inhibitors of apoptosis (IAP) gene have been identified[1]. Among these, a structurally unique member of the IAP proteins, survivin, a Mr~16.500 cytoplasmic protein with a single BIR and no RING finger is unique for its expression in fetal tissue and in a variety of human cancers, but not in non-proliferating adult tissue[2,3]. In addition to its function as an inhibitor of apoptosis, survivin is involved in the regulation of cellular proliferation and angiogenesis in cancer [4,5]. Remarkably, increased survivin expression has been observed in the most common human neoplasm, including oesophageal cancer [6], ovarian carcinoma[7], laryngeal squamous cell carcinoma [8], colorectal carcinoma [5], breast carcinoma[9] and lymphoma[10]. Most of these studies have demonstrated a positive correlation between survivin expression and poor prognosis of the disease, where a multivariate statistical analysis has revealed that survivin expression is an independent prognostic factor for disease progression[6,10-12].

Angiogenesis is an essential step for tumor growth, playing a critical role in tumor invasion and metastasis[13]. Tumors develop angiogenesis by secreting growth factors, to stimulate endothelial migration and proliferation[14,15]. Among these growth factors, VEGF is regarded as the main growth stimulatory factor in the tumor-related angiogenesis[16]. Human VEGF is located on chromosome 6p21.3 and it plays a critical role in the initial phase of tumor growth and neo-vascularisation[17]. In vitro and in vivo experiments have shown that increased VEGF expression is associated with tumor growth and metastasis, whereas inhibition of VEGF expression results in suppression of tumor growth and tumor-induced angiogenesis [18]. Furthermore, A number of studies in various cancer types have confirmed that VEGF over-expression is closely correlated with the presence of metastasis and recurrence and also with poor survival rate of patients[14,19-22], including NPC.

NPC is one of the most common malignancies in certain areas of southern China, South-Asia and North Africa[23,24] and has a dominant clinicopathological behavior of easily invasive and metastasis, which is different from other head and neck cancers [25]. Metastasis to regional lymph node or distant organ and local recurrence are two major causes for treatment failure of this cancer. Currently, the prediction of NPC prognosis is mainly based on the clinical TNM staging, however, NPC patients with the same clinical stage often present different clinical outcomes, suggesting that the TNM stage is insufficient to precisely predict the prognosis of this disease [26-29]. Therefore, it is important to search for novel molecular biomarkers, which can help clinicians improve the prognostic prediction and develop therapeutic intervention for NPC patients. In this study, we assessed the expression of survivin and VEGF in NPC and their correlations to the clinicopathological parameters and overall survival of the patients.

## Materials and methods

### Cases and clinical parameters

For this retrospective study, archival formalin-fixed, paraffin-embedded specimens from 280 primary NPC patients during 1992 ~ 2002 in Sun Yat-Sen University Cancer Center (Guangzhou, China) were recruited. Tumors from the paraffin blocks were underwent tissue microarray construction before immunostaining. Paraffin sections were directly dissected for pathological staining and immunohistochemistry. Tissue sections of NPC blocks were additionally dissected to single slide for apoptosis label in 100 cases of the NPC.

156 patients were diagnosed as differentiated non-keratinized carcinoma (WHO type II) and 124 were diagnosed as undifferentiated carcinoma (WHO type III). The patients were 208 males and 72 females with age range from 14 to 86 years (median age 46). The China NPC 92 staging system was used[30], patients were classified as 13 in stage I, 63 in stage II, 129 in stage III and 75 in stage IV. All patients were treated with standard curative radiotherapy with or without chemotherapy.

### Tissue microarray construction

A fresh section stained with hematoxylin and eosin (HE) was cut from each block. Individual donor blocks were overlaid with the corresponding HE slides and the areas for tissue microarray sampling were marked. Using instrumentation developed at the Mayo Clinic (Beech Instrument Co., USA), two cylindrical cores of 1.0 mm in greatest dimension were removed from each donor paraffin block and transferred to premolded recipient paraffin blocks at defined array positions. Recipient paraffin blocks contained holes of appropriate dimension in a grid pattern, maximally 11 holes wide by 14 holes in length, allowing for 154 tissue cores per block. This construction design permitted multiple blocks with identical array patterns to be constructed simultaneously, serially sectioned at 5 μm onto "charged" glass slides, and stored at 4°C.

### Immunohistochemical staining and scoring

Polyclonal antibody aginst survivin at 1:200 dilution and monoclonal antibody against VEGF in working solution (Santa Cruz Inc., USA) were used in this study. Briefly, tissue sections were de-waxed, incubated in retrieval buffer solution for antigen recovery (DAKO Co., Denmark); incubated with hydrogen peroxide for 10 minutes; blocked with normal serum for 10 minutes; followed by incubation with a primary antibody for 60 minutes and detected by the Catalyzed Signal Amplification Kit (DAKO Co, Carpinteria, USA). Visualization was developed with diaminobenzidine (DAB). Negative controls were carried out by substituting non-immune goat or rabbit serum for the primary antibodies.

The immunostaining results were evaluated and scored independently by two pathologists without knowledge of the clinicopathological outcomes of the patients. Evaluation of the immunostainning results was performed as previously described[31]. Survivin staining results were scored as four levels according to the percentage of cytoplasmic and/or nuclear specific positive tumor cells in 10 high power fields as follows. (-): less than 5%, (+): 5%–25%, (++): 25–50%, (+++): more than 50%. The survivin over-expression was defined as more than 25% tumor cells with positive staining, whereas survivin low-expression was less than 25%.

VEGF staining results were scored as four levels according to the percentage of cytoplasmic and/or membrane positive cells in 10 high power fields as follow. (-): less than 10%, (+): 11%–20%, (++): 21–50%, (+++): more than 50%. The VEGF over-expression was defined as more than 10% tumor cells with positive staining, whereas VEGF low-expression was less than 10%.

### In situ detection of apoptosis and determination of apoptosis index (AI)

Apoptotic cells in 100 cases of NPC were identified by the terminal-deoxynucleotidyl-transferase (TdT)-mediated deoxyuridine triphosphate (dUTP) nick-end-labeling (TUNEL) method (Boehringer Mannheim, Germany). Methods and AI determination were according to previously described[32]. The AI was determined by microscopic evaluation of apoptotic cells or apoptotic bodies in 100 high-power fields (100 × HPFs). The AI was equal to the total number of apoptotic cells or bodies over total number of tumor cells in 100 HPFs.

### Statistical analysis

Data was analyzed using SPSS12.0 software. The associations between survivin, VEGF expression and clinicopathological parameters were assessed by Chi-Square test. The correlation between survivin and VEGF was clarified as Kappa Measure of Agreement test, and the relationship between AI and survivin expression was analyzed by independent samples t test. Kaplan-Meier analysis and log-rank test were used to assess survival rate and to compare the difference of survival rate. Univariate and multivariate regression analyses were performed with the Cox proportional hazards regression model to analyze the independent factors related to prognosis. *P *< 0.05 was considered to be statistically significant.

## Results

### 1. Association between survivin, VEGF expression and clinicopathological parameters in NPC

**Figure 1:**
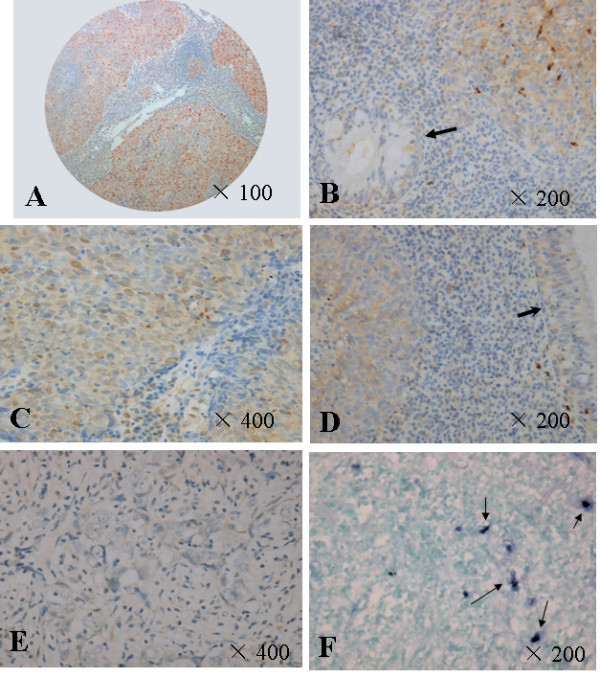
Immunohistochemistry and apoptosis staining of NPC tissue microarray. A. Positive staining of survivin shown under lower power field and B. Cytoplasm positive staining of survivin in the tumor cells and weak staining of the normal epithelium of nasopharynx (arrow directed) C. Strong positive staining of VEGF in tumor cells; D. Cytoplasm positive staining of VEGF in the tumor cells and weak staining of the normal epithelium of nasopharynx (arrow directed); E. Weak staining of survivin in NPC tumor tissues; F. whereas several apoptotic bodies (arrow directed) were observed.

Immunostaining of survivin of the tissue array was representative in Figure 1A. Cytoplasm and/or nucleic positivity for survivin staining were observed in tumor cells, weak expression was observed in normal adjacent nasopharyngeal epithelium (Figure 1B). Totally, 206 cases of the NPC patients were adequate for the final analysis. 74 cases were excluded from this study because of inadequate survivin staining. Among the 206 cases, 44 (21.4%) cases were negative (-), whereas 162 (78.6%) cases were positive, of which, 54 cases (26.2%) were +, 70 cases (34%) were ++, and 38 cases (18.4%) were +++. Over-expression of survivin was observed in 108 (52.4%) out of the 206 NPC tumor tissues, whereas low-expression was observed in 98 cases (47.6%).

Expression of survivin was significantly associated with the clinical stage (*P *= 0.018), T-stage (*P *= 0.004) and lymph node metastasis (*P *= 0.028). No significant association between survivin expression and age, gender, recurrence or distant metastasis was observed (Table 1).

**Table 1:** Correlations between survivin expression and clinicopathological parameters of NPC patients

	Survivin			
	Case	High expression	Low expression	*P *value
	(n = 206)	(n = 108)	(n = 98)	
Age				
<46	95	53	42	0.371
≥46	111	55	56	
Sex				
Male	152	75	77	0.137
Female	54	33	21	
Histological type (WHO)				
II	138	76	62	0.283
III	68	32	36	
T stage				
T1~2	82	33	49	0.004
T3~4	124	75	49	
N stage				
N0	49	19	30	0.028
N1~3	157	89	68	
TNM stage				
I/II	48	18	30	0.018
III/IV	158	90	68	
Recurrence				
Yes	47	27	20	0.433
No	159	81	78	
Metastasis				
Yes	24	13	11	0.856
No	182	95	87	

VEGF positive staining was observed in both membrane and cytoplasm of NPC tumor cells (Figure 1C), whereas weak positive staining was observed in the normal epitheliums (Figure 1D). Totally, 188 cases of the NPC tumors were adequate for the final analysis, 92 cases were excluded from this study because of the VEGF inadequate staining. Among the 188 tumor tissues, VEGF expression was negative (-) in 102 cases (54.3%); whereas 86 cases (45.7%) were positive, of which, 40 cases (21.3%) were +, 20 cases (10.6%) were ++, and 26 cases (13.8%) were +++. VEGF over-expression was observed in 86 (45.7%) out of the 188 cases, whereas VEGF low-expression was observed in 102 cases (54.3%).

Expression of VEGF was significantly associated with clinical stage, recurrence and distant metastasis (*P *< 0.05). No correlation was observed between VEGF expression and gender, age, T stage and lymph node metastasis (*P *> 0.05) (Table 2). Furthermore, over-expression of survivin was positively correlated with over-expression of VEGF in NPC tumors (Kappa = 0.25) (Table 3).

**Table 2:** Relationship between VEGF expression and clinicopathological parameters of NPC patients

	VEGF			
	Case	High expression	Low expression	*P *value
	(n = 188)	(n = 86)	(n = 102)	
Age (years)				
<46	95	41	54	0.472
≥46	93	45	48	
Sex				
Male	147	67	80	0.931
Female	41	19	22	
Histological type (WHO)				
II	124	55	69	0.253
III	64	31	33	
T stage				
T1~2	71	27	44	0.098
T3~4	117	59	58	
N stage				
N0	48	23	25	0.726
N1~3	140	63	77	
TNM stage				
I/II	42	13	29	0.029
III/IV	146	73	73	
Recurrence				
Yes	58	36	22	0.004
No	130	50	80	
Metastasis				
Yes	23	16	7	0.024
No	165	70	95	

**Table 3:** Correlations between survivin and VEGF expression in NPC   tissues

	VEGF		
	High expression	Low expression	Kappa value
Survivin			
High expression	48 (56.5%)	37 (43.5%)	0.25
Low expression	28 (31.1%)	62 (68.9%)	

### 2. Correlation between survivin expression and apoptosis in NPC

**Figure 2:**
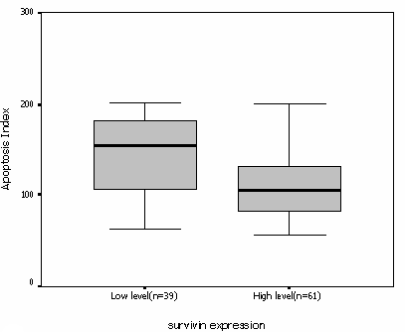
Correlation between survivin expression and apoptosis in NPC, The mean AI in the survivin low-expression cases was significantly higher than that of in survivin over-expression cases (*P *< 0.05).

Total 100 NPC cases were conducted to in situ apoptosis detection. The apoptotic cells and bodies appeared labeled with an intense, dark blue reaction to easily distinguish the survivin low-expressed tumor cells (Figure 1E, 1F). The correlation between AI and survivin expression in NPC was shown in Figure 2. Mean AI value was 128 ± 43.05 (95% CI, 119.6 ~136.7). Significant negative correlation was observed between apoptosis and survivin expression in NPC. The mean AI in the 39 survivin low-expressed cases was 144.7 ± 39.9 (95% CI, 133.4~156.1), which was significantly higher than that of the 61 tumors with survivin highly expressed (111.6 ± 39.8; 95% CI, 100.2~122.9) (T test, *P *< 0.05).

### 3. Associations between survivin, VEGF expression, and survival of NPC patients

**Figure 3:**
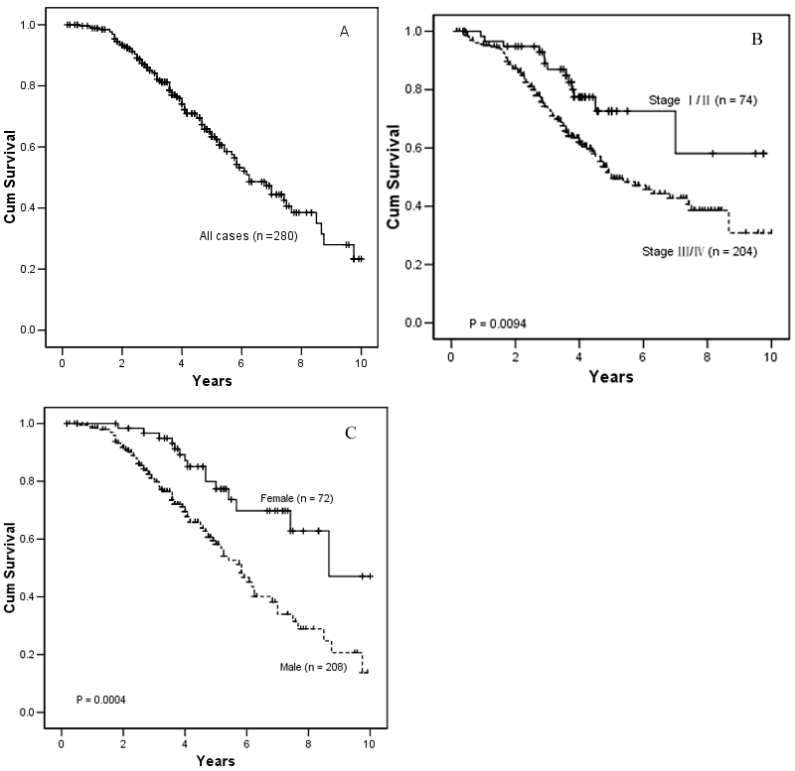
A, Kaplan-Maplan curves for overall survival rates of 280 patients with NPC. B, Kaplan-Maplan curves for overall survival rates of different clinical stage, patients with stage III/IV have a lower survival rate than those with stage I/II (*P *= 0.0094, log rank test). C, Kaplan-Maplan curves for overall survival rates of female and male paitents, male patients present lower survival rate than female paitents (*P *= 0.0004, log rank test)

The 5-year survival rate and 10-year survival rate of the cohort of the 280 NPC patients were 53.4% and 23.2%, respectively (Figure 3A). The 5-year survival rate of the 280 patients with clinical stage I/II and stage III/IV were 72.6% and 49.6%, respectively. There was a statistical significance (*P *= 0.0094) (Figure 3B). When the patient cohort was stratified according to gender, the 5-year survival rate of male patients was significantly lower than that of female patients (47.9% vs. 78.7% respectively, *P *= 0.0004) (Figure 3C). Further analysis revealed that in this group of NPC patients, female patients were more likely to have an earlier stage compared with male patients (37.5% *vs. *23.6%). So the favorable prognosis in female patients may be due to the earlier stage in these NPC patients (data is not shown).

**Figure 4:**
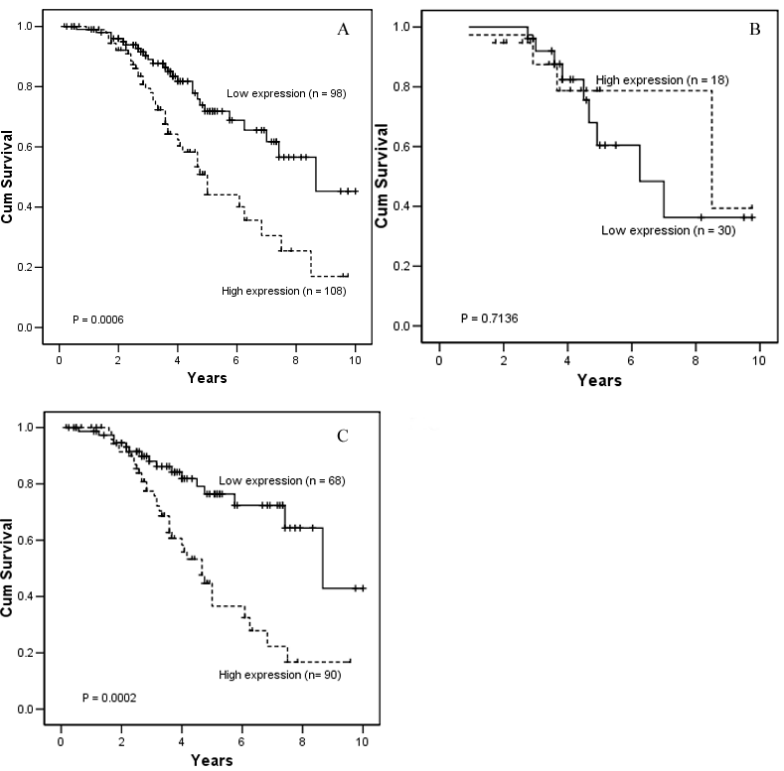
A, Kaplan-Meier curve showing the patients with survivin over-expression have a lower suvivial rate than those with survivin low-expression (*P *= 0.0006). B, no significant difference of 5-year survival rate was found between survivin over-expression and low-expression in NPC patients with early stage (stage I/II, *P *= 0.7136). C, significant difference of 5-year survival rate was found between survivin over-expression and low-expression in NPC patients with advanced stage (stage III/IV, *P *= 0.0002)

According to expression of survivin, the 5-year survival rate of the NPC patients with survivin over-expression was 42.32% (n = 108), which was significantly lower than that in NPC patients with survivin low-expression (70.54%, n = 98, *P *= 0.0006; Figure 4A). Furthermore, when the patient cohort was divided depending on clinical stage and stratified according to tumor expression of survivin, in patients with advanced clinical stage (stages III+IV), the 5-year survival rate of patients with survivin over-expression was significantly lower than that of patients with survivin low-expression (36.51% vs. 73.41%, respectively; *P *= 0.0002; Figure 4C); whereas in the patients with early stage (stages I+II), the difference between survivin over-expression and survivin low-expression was not significant (64.22% vs. 80.46%, respectively; *P *= 0.7136; Figure 4B).

**Figure 5:**
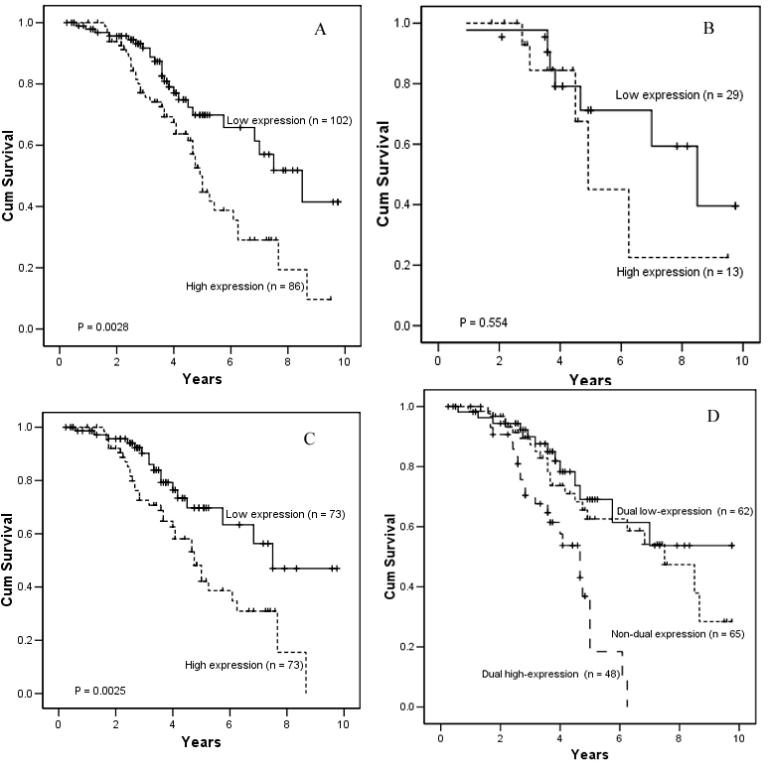
A, Kaplan-Meier curve showing the patients with VEGF over-expression have lower 5-year survival rate than those with VEGF low-expression (*P *= 0.0028). B, Among the patients of stage I/II, no significant difference of 5-year survival rate was found between patients with VEGF over-expression and low-expression (*P *= 0.554). C, Among the patients of stage III/IV, VEGF over-expression group presents lower 5-year survival rate than the VEGF low-expression group (*P *= 0.0025). D, The patients with dual survivin and VEGF over-expression have significant lower 5-year suvivial rate than those with dual survivin and VEGF low-expression group (*P *= 0.0003), and those with non-dual expression group (*P *= 0.001)

When the patient cohort was stratified according to VEGF expression in the tumor cells, the 5-year survival rate of the NPC patients with VEGF over-expression was 40.1% (n = 86), which was significantly lower than that of NPC patients with VEGF low-expression (67.8%, n = 102, *P *= 0.0028; Figure 5A). Furthermore, when the patient cohort was divided by clinical stages and stratified according to expression of VEGF, in NPC patients with advanced clinical stage (stages III+IV), the 5-year survival rate in NPC patients with VEGF over-expression was significantly lower than that of patients with VEGF low-expression (35.03% vs. 65.22%, respectively; *P *= 0.0025; Figure 5C); whereas in early stages NPC (stages I+II), the difference was not significant between NPC patients with VEGF over-expression and those with VEGF low-expression (60.46% vs. 73.17%, respectively; *P *= 0.554; Figure 5B).

When the patient cohort was stratified according to tumor expression of both VEGF and survivin in 175 patients, The 5-year survival rate in 48 NPC patients with survivin and VEGF dual over-expression was significantly lower than that of 62 patients with dual low-expression (18.22% vs. 73.54%, respectively; *P *= 0.0003; Figure 5D).

Using multivariate regression analysis of the entire group of patients, male patients (*P *= 0.026), distant metastasis (*P *= 0.002), VEGF over-expression (*P *= 0.005) and suvivin over-expression (*P *= 0.031) were significantly associated with poor overall survival (Table 4). In addition, each of these four variables was identified as prognostically significant by univariate analysis (Table 5), which demonstrated that the four variables were all independent predictors for the overall survival.

**Table 4:** Multivariate Analysis of Cox Proportional Hazards Model for Overall Survival in NPC patients

Characteristics	Hazard ratio	95%CI	*P*
Sex (male vs. female)	0.463	0.235–0.911	0.026
VEGF (low expression vs. over-expression)	2.205	1.266–3.839	0.005
Survivin (low expression vs. over-expression)	1.834	1.056–3.184	0.031
Metastasis (no vs. yes)	3.598	1.614–8.021	0.002

**Table 5:** Univariate Analysis of Cox Proportional Hazards Model for Overall Survival in NPC patients

Characteristics	Hazard ratio	95%CI	*P*
Age (years, <46 vs. ≥ 46)	1.558	1.044–2.326	0.030
Sex (male vs. female)	0.447	0.264–0.754	0.003
T-stage (T1~2 vs. T3~4)	1.386	0.914–2.101	0.124
N-stage (N0 vs. N1~3)	0.978	0.628–1.524	0.923
Clinical stage (I/II vs. III/IV)	1.817	1.091–3.025	0.022
Recurrence (no vs. yes)	1.560	1.018–2.391	0.041
Metastasis (no vs. yes)	2.023	1.177–3.478	0.011
VEGF (low expression vs. over-expression)	2.112	1.277–3.495	0.004
Survivin (low expression vs. over-expression)	2.132	1.301–3.494	0.003

## Discussion

Multiple genetic pathways control apoptosis, and parts of them may be regulated by survivin gene, but potential mechanism of apoptosis in NPC has not been identified. In this study, we found that the expression of survivin protein in NPC tumor tissues was positively correlated with progression of the patients, especially with primary tumor invasion. Over-expression of survivin may not only play some roles in NPC pathogenesis but also in NPC progression.

In the present study, positive survivin staining was detected in 78.6% (162/206) of NPC cases, with a variable proportion of positive tumor cells. We also identified that survivin over-expression was a significant independent predictor of overall survival (*P *= 0.0006). As mentioned above, survivin expression was previously reported to significantly correlate with poor prognosis in a range of malignant tumors, such as colorectal cancer [12], bladder cancers [33], lymphoma[10], soft-tissue sarcomas[11]. However, so far, there were only few reports about the correlation between survivin expression in NPC and its prognosis. Over-expression of survivin in NPC tumor was reported to be positively correlated with poor prognosis of the patients [34,35]. Our results showed that the 5-year survival rate of the NPC patients with survivin over-expression was significantly lower than that of NPC patients with survivin low-expression, especially in advanced clinical stage (stages III and IV) NPC patients. Our large cases study was a powerful proof for previous conclusion that detection of survivin protein expression in the tumor tissue was helpful for predicting prognosis of NPC patients. Moreover, our results also showed over-expression of survivin was a specific poor prognostic factor for advanced stage NPC patients.

VEGF is considered to be the most cardinal vascular growth factor prompting tumor angiogenesis. Accumulating studies so far suggest that over-expression of VEGF has an positive influence on tumor growth, metastatic potential, failure to radiotherapy and poor prognosis[21,36-40]. In head and neck squamous cell carcinomas, VEGF is a marker of tumor invasion and metastasis [41]. In NPC, the level of serum-VEGF has been reported to be significantly elevated in the patients, which was correlated with primary tumor progression, local recurrence or distant metastasis, and poor prognosis[42,43]. In this large sample study, we found that VEGF over-expression in NPC tumor was significantly correlated with advanced clinical stage, local recurrence and distant metastasis of the disease, and poor prognosis of the patients, which was in accordance with previous reports in NPC and other malignancies [14,21,38-40,44]. Further survival analysis revealed that over-expression of VEGF in the NPC tumor tissue was significantly correlated with decreased 5-year survival rate, especially in patients with advanced clinical stage (stages III and IV), the 5-year survival rate in patients with VEGF over-expression was significantly worse than that of patients with VEGF low-expression. This finding agreed with the results of previous studies that increased tumor VEGF expression was related to poor prognosis of NPC patients [45-47]. These results suggest that VEGF may play an important role in local recurrence and metastasis through induction of angiogenesis in NPC, and VEGF expression is a valuable prognostic marker for prognosis prediction in advanced stage NPC patients.

Furthermore, we found that over-expression of survivin was significantly positively correlated with over-expression of VEGF in NPC. Importantly, the patients with dual over-expression of both survivin and VEGF presented a worse prognosis than those with dual low-expression. The 5-year survival rate of NPC patients with survivin and VEGF dual over-expression was significantly lower than that of patients with dual low-expression. Multivariate analysis indicated that both survivin and VEGF expression in NPC tumor tissues were strong independent factors for poor prognosis of NPC patients (Table 5). These results indicated that co-analysis of VEGF and survivin protein expression in NPC tumor tissues was more valuable for prognosis evaluation of NPC patients.

To our knowledge, the present data firstly provide a compelling case confirming a correlation among the survivin expression, VEGF expression and the survival of NPC patients. As EBV infection was one of the important etiology factors of NPC, and EBV encoded oncoprotein LMP1 was frequently detected in NPC tumor tissues[32], several studies reported that in NPC carcinogenesis, LMP1 up-regulate survivin and VEGF expression directly or indirectly [48-50]. Previous studies also reported that survivin as one of the target genes induced by VEGF in endothelium, which was associated with prominent up-regulation of survivin in newly formed blood vessels during angiogenesis in vivo [51]. Taking together, we assume that in NPC, LMP1 induction of over-expression of suvivin and VEGF, enhance tumor angiogenesis tumor cell infiltration and invasion, and inhibit apoptosis of tumor cells, result in poor prognosis. However, further investigation about this is needed.

## Conclusion

Currently, clinical TNM stage is insufficient to predict the NPC prognosis, patients of the same clinical stage often show different clinical course. This study demonstrates that expression of survivin is consistent with expression of VEGF in NPC and both are closely correlated with poor prognosis of NPC. We suggest that detection of survivin and VEGF expression might aid in the stratification of patients with NPC, especially divided advanced stage NPC into two separate groups whose prognosis was significantly different. In advanced stage NPC patients, Survivin and VEGF high expression render tumor cell resistant to the conventional therapies including radiotherapy and/or chemotherapy and may need more systemic treatment or targeted antagonists of survivin and VEGF therapy.

## List of abbreviations

NPC, nasopharyngeal carcinoma; 

EBV, Epstein-Barr virus; 

VEGF, vascular endothelial growth factor; 

AI, apoptosis index; 

IAP, inhibitors of apoptosis.

## Competing interests

The author(s) declare that they have no competing interests.

## Authors' contributions

LYH, HCF carried out the cases collection, results analysis, drafted the manuscript. SQ, HMY and HJH carried out the immunohistochemistry staining work. XD helped the in situ TUNEL labeling works. ZYX and SJY conceived of the study, participated in its design and coordination and helped to draft the manuscript. All authors read and approved the final manuscript.
